# Indirect feedback hinders explicit sensorimotor adaptation

**DOI:** 10.1098/rspb.2025.1407

**Published:** 2025-07-30

**Authors:** Yifei Chen, Sabrina Abram, Richard B. Ivry, Jonathan S. Tsay

**Affiliations:** ^1^ Department of Psychology, Princeton University, Princeton, NJ, USA; ^2^ Department of Psychology, University of California, Berkeley, CA, USA; ^3^ Department of Neuroscience, University of California, Berkeley, CA, USA; ^4^ Department of Psychology, Carnegie Mellon University, Pittsburgh, PA, USA

**Keywords:** motor learning, declarative memory, implicit learning, explicit learning, symbolic feedback

## Abstract

Motor adaptation—the process of reducing motor errors through feedback—is an essential feature of human competence, allowing us to move accurately in dynamic and novel environments. Adaptation typically results from direct sensory feedback, with most learning driven by visual and proprioceptive feedback that arises with the movement. In humans, motor adaptation can also be driven by indirect numerical feedback. In the present study, we examine how implicit and explicit components of motor adaptation are modulated by indirect numerical feedback. We conducted three reaching experiments involving over 400 human participants to compare direct sensory feedback and indirect numerical feedback using a task in which both types of learning processes could be present (experiment 1) or tasks in which learning was expected to be limited to only an explicit process (experiments 2 and 3). Adaptation with indirect feedback was dominated by explicit strategy use, with minimal evidence of implicit recalibration. When matched for information content, adaptation to both rotational and mirror-reversal perturbations was slower with indirect feedback than with direct feedback, due to increased random and systematic exploration. These results suggest that the nature of feedback shapes strategic discovery, offering new insights into how feedback type influences the mechanisms of sensorimotor learning.

## 1. Introduction

Motor adaptation—the process of reducing motor errors through feedback—enables us to flexibly move in dynamic and novel environments [1–3]. For example, motor adaptation enables a golfer to adjust her swing to accommodate changes in terrain and wind conditions, and similarly allows a marathon runner to maintain consistent force output despite increasing muscle fatigue.

Motor adaptation is not a singular process but instead entails the operation of multiple learning processes. Paralleling the memory literature, one broad distinction can be made between processes that are under conscious control and those that operate outside awareness: whereas explicit strategy use can allow the agent to reduce performance errors in a volitional and conscious manner [4–6], implicit recalibration keeps our movements finely calibrated in an automatic and subconscious manner. Indeed, the interplay of explicit and implicit processes in motor adaptation has been the focus of many studies over the past decade [7].

Implicit and explicit learning processes exhibit distinct properties. Whereas implicit recalibration is a relatively rigid process, capable of producing limited, incremental changes in behaviour, explicit strategy use is remarkably flexible, capable of producing rapid changes that can be quite dramatic [8,9]. Moreover, while implicit recalibration is highly sensitive to the timing of the feedback and is severely attenuated when the feedback is delayed, strategy-based learning is minimally impacted by manipulations of the timing of the feedback [10–16].

Learning in most contexts is based on visual and proprioceptive feedback that arises during the movement. These forms of sensory feedback convey motor errors through *direct* experience, offering a clear mapping between the error and the required correction: the archer sees their shot off to the left of the bullseye or the guitarist feels their fingers misplaced for the desired chord. In humans, learning can also be driven by *indirect* feedback, where abstract information must be cognitively transformed into movement corrections. For example, the golfer taking a short cut over the trees might hear a collective groan from the crowd and be fearful that their shot has landed in the pond just in front of the green. Or a blindfolded dart thrower, when aiming for the ‘15’, would know they were too high when informed that the dart was in the ‘13’ slot. While direct feedback can be exploited by both implicit and explicit adaptation processes [17–20], it is unclear if the same holds for indirect feedback.

In this study, we posed two questions. First, what learning processes are elicited by indirect feedback? We revisited this question because prior studies using indirect feedback often used tasks that did not clearly dissociate implicit and explicit learning processes [21–27]. For example, previous studies did not include manipulations such as asking participants to verbally report where they aimed to measure strategy use [4], or instruct participants to forgo strategy use and reach directly to the target when measuring the aftereffect once the perturbation was removed [28,29]. The only study that has used such methods found that indirect feedback was dominated by explicit strategy use [30]. We aimed to replicate this finding, scaling from an initial sample of approximately 20 to a substantially larger sample of approximately 180.

Second, is adaptation under indirect feedback as efficient as under direct feedback? This question has not been clearly answered, given that previous studies have not equated error information conveyed between direct and indirect feedback: while direct feedback conveys both error direction and magnitude, indirect feedback typically provides only one or the other [27,30]. Furthermore, previous studies have not characterized the pattern of errors in detail, leaving it unclear *why* feedback type affects adaptation. Is the difference driven by increased random exploration, systematic exploration or both?

To address these questions, we conducted three motor adaptation experiments involving 415 human participants. In experiment 1, we manipulated the size of the rotational perturbation (30°, 60° and 90°), with movement feedback conveyed indirectly through a numerical score. If indirect feedback elicits implicit recalibration, we expect to observe large and robust aftereffects across all three perturbation sizes, a persistent change in hand angle away from the target after the perturbation is removed and participants are instructed to reach directly towards to the target. Moreover, similar to what is observed with direct feedback, the magnitude of this aftereffect should be similar for all three perturbation sizes. Conversely, if indirect feedback is dominated by explicit strategy, we expect learning to scale with perturbation sizes but result in minimal aftereffects.

In experiments 2 and 3, we examined whether the abstract nature of indirect numerical feedback, compared with the concrete nature of direct sensory feedback, impacts the discovery of a successful explicit strategy. To address this question, we delayed the presentation of feedback to isolate explicit strategy, allowing us to contrast the same learning process’s responses to direct and indirect feedback while tightly matching the error information conveyed (i.e. magnitude and direction). We also applied model-based analyses to examine whether feedback type differentially influenced systematic exploration—reflected in distinct performance peaks—and random exploration, indicated by broader, uniform distributions across the workspace.

## 2. Methods

### (a) Participants and apparatus

A total of 415 participants completed the study (female: 216; male: 174; other: 25; 24.74 ± 0.17 years old). Participants were recruited on a web-based crowdsourcing platform (www.prolific.com) and were compensated at $12.00 per hour. We limited recruitment to participants who (i) have a minimum 95% approval ratings and (ii) spoke English as their first language. While handedness was not a recruitment criterion (right-handed: 362; left-handed: 46; ambidextrous: 9), our key findings remained robust even when the analyses were limited to right-handed participants (see electronic supplementary material, figure S3).

The sample size in each experiment was informed by similar web-based motor adaptation studies [14,31,32], as well as considerations for counterbalancing. Note that our sample size is significantly greater than comparable in-lab motor adaptation studies (approx. 40 participants) [22,27,30].

The experiment was created using the OnPoint platform, a package for running customized online motor learning experiments with JavaScript. Participants completed the web-based experiment through an internet browser with their own devices (trackpad: 331; optical mouse: 82; trackball: 2). Our past online studies have shown that neither the type of browser nor the type of pointing device significantly impacts performance [33]. The size and position of the visual stimuli were dependent on the individual’s monitor size. For ease of interpretation, all stimulus parameters detailed below were based on an average 13-inch computer monitor.

### (b) General procedure

For each trial, participants were asked to position their cursor, a white dot (diameter = 0.4 cm), inside a white starting ring (diameter = 0.5 cm), which was located at the centre of the screen. Once the cursor was moved inside, the starting ring was filled. After holding the cursor inside the starting ring for 500 ms, the cursor was blanked, eliminating visual feedback and a blue circular target (diameter = 0.4 cm) appeared along an invisible ring with a radius of 8 cm relative to the starting ring. The blue target could appear in one of the three target locations on the invisible ring. The sequence of target locations was presented pseudo-randomly within each movement cycle (i.e. 1 movement cycle = 3 reaches: 1 reach to each target location).

### (c) Feedback

Feedback was provided immediately (experiment 1) or 800 ms after movement termination (experiments 2−3) and remained visible for 1 s. Indirect feedback was provided through a numerical score. How the numerical score was calculated varied between experiments. In experiment 1, the score conveyed only error magnitude, the absolute distance between the participant’s movement endpoint and the target, with the score rounded to the nearest integer. To make the scores easier for the participants to understand, we normalized the range of scores from a minimum of 0 points (hand angle 180° away from the target) to a maximum of 100 points (hand angle at the bullseye of the target). In experiments 2 and 3, indirect numerical feedback conveyed both error magnitude and direction. Here, the score could range from −180° to 179°. As such, 0 was the best possible score in these experiments, with negative values indicating a counterclockwise error and positive values a clockwise error (e.g. a score of +60 signified that the endpoint cursor location was 60° clockwise from the target location).

We also included direct feedback conditions in experiments 2 and 3. Direct feedback was provided through a white cursor that appeared on the ring, indicating the participant’s hand position when the movement amplitude was 8 cm (veridical feedback trials) or displaced from that hand position by the visual perturbation (rotation trials).

### (d) Experiment 1

Participants (*n* = 184; female: 93; male: 77; other: 14; 25.06 ± 0.29 years old) were randomly assigned to one of the three perturbation groups (30° rotation: 77; 60° rotation: 51; 90° rotation: 56). Two participants whose average hand angles were five standard deviations away from the group means were excluded. We counterbalanced the direction of the perturbation (clockwise or counterclockwise) across participants within each perturbation group. There were three target locations: 30° (upper-right quadrant), 150° (upper-left quadrant) and −90° (straight down) (see electronic supplementary material, figure S1a).

Movement feedback was always provided indirectly in the form of a numerical score that ranged between 0 and 100 points. There were three blocks: baseline veridical feedback (30 trials; 10 cycles), rotated feedback (150 trials; 50 cycles) and no-feedback aftereffect (30 trials; 10 cycles). In the baseline block, participants were familiarized with the basic reaching procedure and the indirect feedback. Participants were provided the following instructions: ‘Move directly to the target. You will be rewarded based on your accuracy (max score = 100 points).’ In the perturbation block, the score was based on the rotated (30°, 60° or 90°) endpoint hand position. Thus, to get 100 points on a trial, a participant in the 60° clockwise perturbation group would have to move 60° counterclockwise to the target. Participants were instructed: ‘Move somewhere away from the target. Find the movement direction that yields 100 points.’ In the aftereffect block, the perturbation was removed. Participants were given the following instructions: ‘Move directly to the blue target, and do not aim away from the target’. There was no feedback presented during the aftereffect block.

### (e) Experiment 2

Participants (*n* = 110; female: 58; male: 46; other: 6; 24.33 ± 0.31 years old) were randomly assigned to one of the two groups that differed in terms of feedback type (direct: 53; indirect: 57). For both groups, the feedback provided vectorial information (magnitude and direction). Importantly, the feedback was presented 800 ms after the hand had reached the target amplitude—a manipulation that greatly attenuates or even eliminates implicit recalibration [10,11]. In this way, we sought to focus on comparing explicit strategy use in response to direct or indirect feedback. We counterbalanced the direction of the perturbation (clockwise or counterclockwise) across participants within each feedback group. The target locations were the same as experiment 1 (see electronic supplementary material, figure S1*a*).

There were three experimental blocks: baseline veridical feedback (30 trials; 10 cycles), delayed 60° rotated feedback (150 trials; 50 cycles) and no-feedback aftereffect (30 trials; 10 cycles). Unlike experiment 1, we introduced 12 instruction familiarization trials at the beginning of the baseline block to ensure that participants fully understood the error information conveyed by the feedback. During the first six familiarization trials, the following instructions accompanied the feedback display: ‘You missed the target by X° in the X direction’ (direct group) or ‘You missed the target by X° in the X direction; a positive score signifies a clockwise error and a negative score signifies a counterclockwise error’ (indirect group). Participants advanced to the next trial by pressing the space bar. In the final six familiarization trials, participants were required to report the direction of their error after the feedback was presented (press ‘a’ for a clockwise error; press ‘b’ for a counterclockwise error). The experiment was terminated if participants provided inaccurate responses for more than two of these six trials.

### (f) Experiment 3

Participants (*n* = 121; female: 65; male: 51; other: 5; 24.62 ± 0.30 years old) were assigned to one of the two groups receiving direct (*n* = 50) or indirect (*n* = 71) feedback. The feedback was provided in the same manner as experiment 2. We designed experiment 3 to contrast learning performance in response to indirect and direct feedback conveying a mirror-reversal perturbation. Specifically, the endpoint hand position was mirror-reversed across either the horizontal or vertical axis (reversal axes counterbalanced among participants). For example, in the horizontal mirror condition, if participants reached to the 30° target, their endpoint hand position would be reflected to the −30° location, resulting in a 60° error. Similarly, in the vertical mirror condition, if participants reached to the −120° target, their endpoint hand position will be reflected to the −60° location, also resulting in a 60° error. Note that, unlike a rotational perturbation, re-aiming in the opposite direction of the error (direct or indirect) would increase the error in response to the mirror transformation.

The target locations were dependent on the mirror-reversal axis to maintain a consistent 60° error if participants move directly to the target. Specifically, participants experiencing a horizontal mirror reversal moved to targets located at 30°, 150° and −150°, or to targets located at 30°, 150° and −30° (counterbalanced across participants). Likewise, participants experiencing a vertical mirror reversal moved to targets located at 60°, 120° and −120°, or to targets located at 60°, 120° and −60° (counterbalanced across participants) (see electronic supplementary material, figure S1*b*).

There were three blocks: baseline veridical feedback (30 trials; 10 cycles), delayed mirror-reversed feedback (150 trials; 50 cycles) and no-feedback aftereffect (30 trials; 10 cycles). To ensure participants fully understood the feedback provided, we incorporated an instruction familiarization block similar to that of experiment 2.

### (g) Data analysis

We focused our analyses on the hand position data recorded when the movement amplitude reached the target radius. These data were used to calculate our main dependent variable, hand angle, the difference between the hand position and the target. Hand angles across different perturbation directions were flipped such that positive hand angles always signified changes in heading angle that nullifies the perturbation. Hand angles were also baseline subtracted to correct for small idiosyncratic movement biases [34,35]. Baseline performance included all 10 cycles of the baseline veridical feedback block (trials 1 – 30). Early adaptation was operationally defined as the first 10 cycles of the perturbation block (trials 31–60) and late adaptation was defined as the last 10 cycles of the perturbation block (trials 151–180). Aftereffect performance included all 10 cycles of the aftereffect block (trials 181–210). We also used a continuous performance measure to compare the groups, implementing a cluster-based permutation test on the hand angle and reaction time data [36–38] (see electronic supplementary material, Supplemental Methods I).

We performed a subgroup analysis exclusively on ‘learners’ [10,11,39]. Using a pair of liberal criteria, learners were defined as participants whose (i) hand angle was greater than 20% of the perturbation (e.g. greater than 12° of the 60° perturbation) and (ii) demonstrated a significant change in hand angle in the direction that correctly counteracts the perturbation during late adaptation (one-tailed paired *t*-tests between baseline and late adaptation, *t >* 0 and *p <* 0.05). Two participants who constantly aimed towards the opposite direction of the target were manually removed from ‘learners’ by visual inspection. This analysis yielded 112 learners in experiment 1 (proportion of learners: 61%), 76 learners in experiment 2 (69%) and 75 learners in experiment 3 (62%).

To characterize and compare exploration strategies during early adaptation, we fit a mixture of von Mises distributions with a uniform component to the hand angle data to capture both systematic and random exploration [40,41]. Group differences in the hand angle distributions were quantified using Jensen–Shannon divergence (JSD), with significance determined by permutation testing (see electronic supplementary material, Supplemental Methods II).

## 3. Results

### (a) Experiment 1: motor adaptation in response to indirect feedback is dominated by explicit strategy use

**Figure 1.**
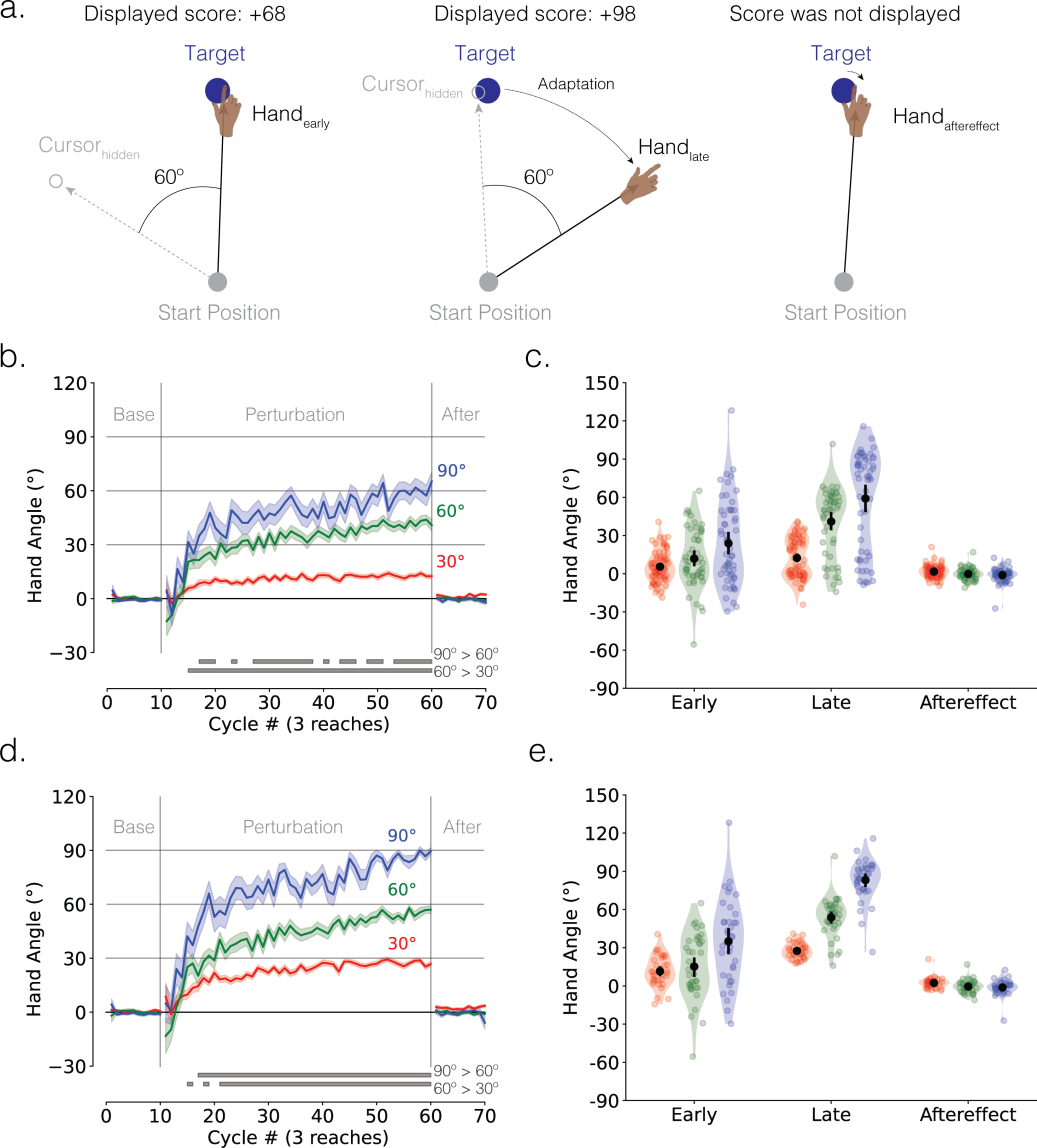
Motor adaptation in response to indirect feedback is dominated by strategy use. (a) Schematic of the web-based rotational perturbation task in experiment 1. An example of the 60° counterclockwise rotation is provided. Participants are instructed to reach in the direction that maximized points (i.e. 100 points). The left, middle and right panels display a representative trial from the early adaptation, late adaptation and aftereffect phases, respectively. (b) Mean time courses of hand angle (*n* = 184; 30°: 77 participants; 60°: 51 participants; 90°: 56 participants). Colours denote different perturbation groups (red = 30°; green = 60°; blue = 90°). Shaded error denotes s.e.m. Each movement cycle includes three trials (1 reach to each of the three targets). Grey horizontal lines at the bottom indicate clusters showing significant group differences. (c) Mean hand angles during early adaptation (i.e. first 10 cycles of the perturbation block), late adaptation (last 10 cycles of the perturbation block), and aftereffect phases (10 cycles of the aftereffect block). Black line denotes mean ± 95% confidence interval. (d) Mean time courses and (e) mean hand angles of learners (*n* = 112; 30°: 35 learners; 60°: 39 learners; 90°: 38 learners).

We presented unsigned indirect numerical feedback in experiment 1, varying the size of the rotational perturbation (30°, 60° and 90°) in a between-group design (figure 1*a*). Feedback was limited to a number ranging from 0 (cursor moved in the opposite direction of the target) to 100 (cursor landed on target). We included a no-feedback aftereffect block in which we instructed the participants to reach ‘directly to the target’. The cardinal signature of implicit recalibration is a residual deviation in hand angle during the aftereffect block, with the magnitude of the effect similar across different perturbation sizes. Signatures of explicit strategy use include (i) the scaling of adaptation across these large perturbation sizes, considering that implicit recalibration should have already saturated by 30° [8,18] and (ii) an immediate and large change in hand angle back towards the target at the start of the aftereffect block.

**Figure 2.**
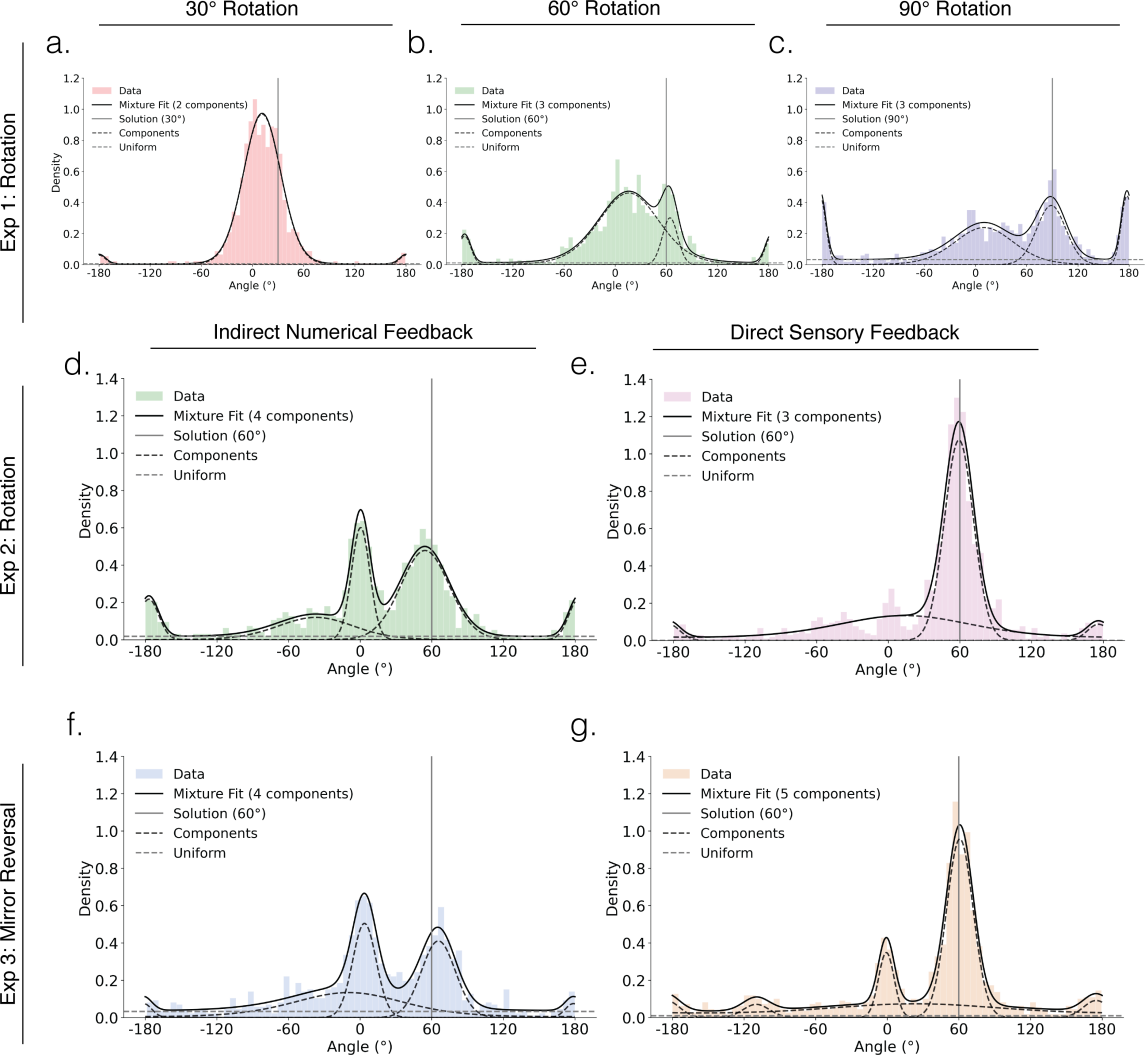
Indirect numerical feedback leads to greater random and systematic exploration than direct sensory feedback. The density plots show hand angle distributions during early adaptation (first 10 cycles of the perturbation block) for learners in the three experiments. Eexperiment 1: (a) 30° (red), (b) 60° (green) and (c) 90° (blue) perturbation groups. Experiment 2: (d) indirect (green) and (e) direct (purple) feedback. Experiment 3: (f) indirect (royal blue) and (g) direct (orange) feedback. Solid grey lines show correct solution to the perturbation. Solid black lines indicate the overall fits from the von Mises mixture model; dashed lines show individual components. The uniform component (grey dashed line) captures random exploration.

Overall, participants improved their scores over the course of the perturbation block, exhibiting a change in hand angle away from the target (figure 1*b*). Even though the mean hand angle for each groups fell considerably short of optimal performance during late adaptation, the learning functions scaled with the size of the rotation. Notably, there was a large change in hand angle at the start of the aftereffect block and minimal evidence of a residual aftereffect in all three groups. Together, these results are consistent with the hypothesis that adaptation in response to indirect numerical feedback is reliant on explicit re-aiming, with minimal evidence of any implicit recalibration.

These observations were confirmed in a series of statistical tests. In terms of the tests of strategic re-aiming, mean hand angle throughout the perturbation block exhibited a main effect of perturbation size (cluster-based *F*-test for a main effect of perturbation size, *F*_score_ = 1579.50, *p*_perm_<0.05, *d* = 0.11). Post hoc cluster-based *t*-tests showed that learning was greatest for 90° and smallest for 30° (grey solid lines in figure 1*b*: 90° > 60°: *t*_sum_>4.72, *p*_perm_<0.05, *d* > 0.24; 60° > 30**°**: *t*_sum_ = 302.25, *p*_perm_<0.05, *d* = 0.75).

We observed similar scaling effects in reaction time, with reaction time being longest for the 90**°** group and shortest for the 30**°** group (electronic supplementary material, figure S4a; 90° > 60°: *t*_sum_ > 4.13, *p*_perm_< 0.05, *d* > 0.22; 60° > 30°: *t*_sum_>5.59, *p*_perm_< 0.05, *d* > 0.27). The scaling of reaction time has been taken to indicate that both the discovery of an explicit strategy becomes more computationally demanding as the perturbation size increases [42–44]. In contrast, varying the perturbation size across the range used in experiment 1 has negligible effects on both the learning functions and reaction time in tasks that isolate implicit recalibration (see [7,45,46]).

Further evidence of strategy use is given by the observation that all three groups showed a significant drop in hand angle from late adaptation to aftereffect block (figure 1*c*; paired *t*-tests comparing aftereffect versus late adaptation: 30°: −10.69 ± 1.69°, *t*(76) = −6.59, *p* < 0.001, *d* = −0.75; 60°: −41.16 ± 3.82°, *t*(50) = −10.99, *p* < 0.001, *d* = −1.54; 90°: −60.12 ± 5.22°, *t*(55) = −11.02, *p* < 0.001, *d* = −1.47). Indeed, we observed either minimal or absent aftereffects across the three perturbation groups (figure 1*c*; paired *t*-tests comparing aftereffect versus baseline, 30°: 1.70 ± 0.55°, *t*(76) = 3.20, *p* = 0.002, *d* = 0.37; 60°: −0.18 ± 0.64°, *t* (50)= −0.28, *p* = 0.782, *d* = −0.04; 90°: −1.09 ± 0.74°, *t*(55) = −1.88, *p* = 0.07, *d* = −0.25). Thus, the data suggest that the error information conveyed by indirect numerical feedback is not sufficient to engage the process underlying implicit recalibration, similar to the findings of Butcher & Taylor [30]. Note that the absence of aftereffect is not a peculiarity of conducting remotely experiments over the web, as many web-based studies have elicited robust implicit recalibration in response to a rotational perturbation [33,39,47].

As noted above, late adaptation for all groups fell considerable short of optimal performance (paired *t*-tests comparing late adaptation versus baseline: 30°: 12.40 ± 1.75°, *t*(76) = 7.33, *p* < .001 , *d* = 0.84; 60°: 40.98 ± 3.78°, *t*(50) = 11.06, *p* < 0.001, *d* = 1.55; 90°: 59.02 ± 5.15°, *t*(55) = 10.93, *p* < 0.001, *d* = 1.46). Inspection of individual data indicated that there were several participants in each group who exhibited minimal improvement, with mean hand angles remaining close to baseline (i.e. towards the target; figure 1*c*; 30°: 55% non-learners; 60°: 24% non-learners; 90°: 32% non-learners; see electronic supplementary material, figure S5 for representative non-learners). While the presence of non-learners may point to a general performance issue (e.g. failure to attend to the task), it may also highlight how learning in response to indirect feedback is unlikely to be automatic and implicit, but instead, explicit and computationally demanding [48,49].

Given that each group appears to be composed of ‘learners’ and ‘non-learners’, we repeated the key analyses with only the data from the ‘learners’ (see §2 for inclusion criteria). In this restricted, post hoc analysis, the core observations noted above were even more striking, with late adaptation approaching the full perturbation (paired *t*-tests against the baseline: 30°: 27.52 ± 1.04°, *t*(34) = 26.54, *p* < 0.001, *d* = 4.49; 60°: 53.76 ± 2.43°, *t*(38) = 22.10, *p* < 0.001, *d* = 3.54; 90°: 83.10 ± 2.59°, *t*(37) = 32.10, *p* < 0.001, *d* = 5.21). The change in hand angle during late adaptation scaled with the size of the perturbation (figure 1*d*; 90° > 60°: *t*_sum_ = 209.99, *p*_perm_ < 0.05, *d* = 0.63; 60° > 30°: *t*_sum_ > 5.00, *p*_perm_ < 0.05, *d* > 0.32), as did reaction times (electronic supplementary material, figure S4d; 90° > 60°: *t*_sum_ > 6.98, *p*_perm_ < 0.05, *d* > 0.30; 60° > 30**°**: *t*_sum_ > 14.12, *p*_perm_ < 0.05, *d* > 0.34).

Perhaps most interesting, the aftereffects remained negligible in the restricted analysis, being significantly different from zero only in the 30° group (figure 1*e*; 30°: 2.36 ± 0.74°, *t*(34) = 3.22, *p* = 0.003, *d* = 0.54; 60°: −0.49 ± 0.79°, *t*(38) = −0.62, *p* = 0.536, *d* = −0.10; 90°: −1.20 ± 0.97°, *t*(37) = −1.70, *p* = 0.100 , *d* = −0.28). It is unclear whether this 2° aftereffect in the 30° group is the result of implicit recalibration [50] or a small use-dependent bias caused by repeated movements away from the target [51–55].

We next asked whether perturbation size influenced how participants *discovered* a successful strategy. To test this, we examined hand angle distributions during early adaptation, the exploratory phase. We restricted this analysis to the learners. The data revealed three distinct patterns. First, all groups showed multimodal distributions (table 1), suggesting that participants systematically explored multiple distinct directions. This supports the idea that participants were deliberately reasoning about and testing distinct action–outcome hypotheses to discover an optimal strategy (figure 2*a–c*), rather than merely reducing error through gradual trial and error [7].

**Table 1.** Exploration across perturbation types and feedback types. This table summarizes von mises mixture model parameters from learners during early adaptation (experiments 1−3), grouped by perturbation and feedback types. Reported parameters include component means (*μ*), concentration (*κ*), weights (*w*) for each von Mises component and the weight (*w*) of the uniform (random) component. The peaks of the von Mises distribution reflect systematic exploration, while the uniform component captures random exploration.

experiment	perturbation	feedback	component	mean *(μ)*	concentration *(κ)*	weight *(*w*)*
1	30° rotation	indirect	von Mises	11.4°	6.9	0.94
			von Mises	−179.7°	58.4	0.02
			uniform	—	—	0.04
	60° rotation	indirect	von Mises	16.1°	2.7	0.75
			von Mises	64.3°	40.5	0.12
			von Mises	−176.6°	62.5	0.06
			uniform	—	—	0.07
	90° rotation	indirect	von Mises	10.0°	2.9	0.38
			von Mises	88.8°	11.3	0.29
			von Mises	177.5°	74.0	0.13
			uniform	—	—	0.21
2	60° rotation	direct	von Mises	59.2°	22.3	0.57
			von Mises	13.6°	1.0	0.39
			von Mises	176.1°	38.1	0.04
			Uniform	—	—	0.00
	60° rotation	Indirect	von Mises	54.3°	8.0	0.43
			von Mises	−37.1°	3.3	0.17
			von Mises	0.4°	54.8	0.20
			von Mises	−177.0°	47.8	0.08
			uniform	—	—	0.11
3	mirror	direct	von Mises	−0.8°	77.1	0.10
			von Mises	−109.7°	27.5	0.03
			von Mises	60.8°	26.4	0.47
			von Mises	23.6°	0.5	0.29
			von Mises	175.1°	28.5	0.04
			uniform	—	—	0.06
	mirror	indirect	von Mises	3.7°	33.9	0.22
			von Mises	65.6°	16.3	0.26
			von Mises	−9.5°	1.6	0.30
			von Mises	178.7°	71.5	0.02
			uniform	—	—	0.21

Second, the groups showed consistent peaks around 0° (directly to the target), their unique solutions (30°, 60° or 90°), and interestingly, 180° (opposite the target). The 0° peak probably reflects a default response before forming a new strategy at the solution while the 180° peak suggests that reaching in the opposite direction may be a common action-outcome hypothesis, regardless of the perturbation size. There is also a uniform component in the distributions. This may indicate some degree of random exploration, although it could also reflect hypotheses that were tested but not prominently sampled.

Third, as shown in figure 2, hand angle distributions varied with perturbation size, as quantified by Jensen–Shannon divergence (30° versus 60° = 0.635, *p* = 0.169; 60° versus 90° = 0.648, *p* = 0.613; 30° versus 90° = 0.849, *p* = 0.001). As perturbation size increased, participants became more likely to reach in the opposite direction of the target (figure 2*a*; 30°: *μ* = −179.7°, κ = 58.4, *w* = 0.02; figure 2*b*; 60°: *μ* = −176.6°, κ = 62.5, *w* = 0.06; figure 2*c*; 90°: *μ* = 177.5°, κ = 74.0, *w* = 0.13). Participants also showed increased random exploration, as indicated by greater weight on the uniform distribution component (figure 2*a*; 30°: *w* = 0.04; figure 2*b*; 60°: *w* = 0.07; figure 2*c*; 90°: *w* = 0.21). In contrast, reliance on the default strategy of reaching near the target declined with increasing perturbation size (figure 2*a*; 30°: *μ* = 11.4°, κ = 6.9, *w* = 0.94; figure 2*b*; 60°: *μ* = 16.1°, κ = 2.7, *w* = 0.75; figure 2*c*; 90°: *μ* = 10.0°, κ = 2.9, *w* = 0.38). Overall, these data suggest that larger perturbation sizes prompted participants to engage in both systematic and random exploration of alternative action-outcome hypotheses.

Together, the results of experiment 1 highlight that motor adaptation in response to indirect numerical feedback was driven by strategy use. Moreover, larger perturbations were associated with greater systematic and random exploration.

### (b) Experiment 2: motor adaptation in response to a rotational perturbation is decelerated by indirect compared with direct feedback

The results of experiment 1 raise the question of whether the processes underlying strategy discovery differ depending on whether they are triggered by indirect numerical or direct sensory feedback. Previous studies comparing direct and indirect feedback have *not* matched the error information (direction and magnitude) conveyed by these different types of feedback [21–27,30,56,57]. We set out to fill this gap in experiment 2.

**Figure 3.**
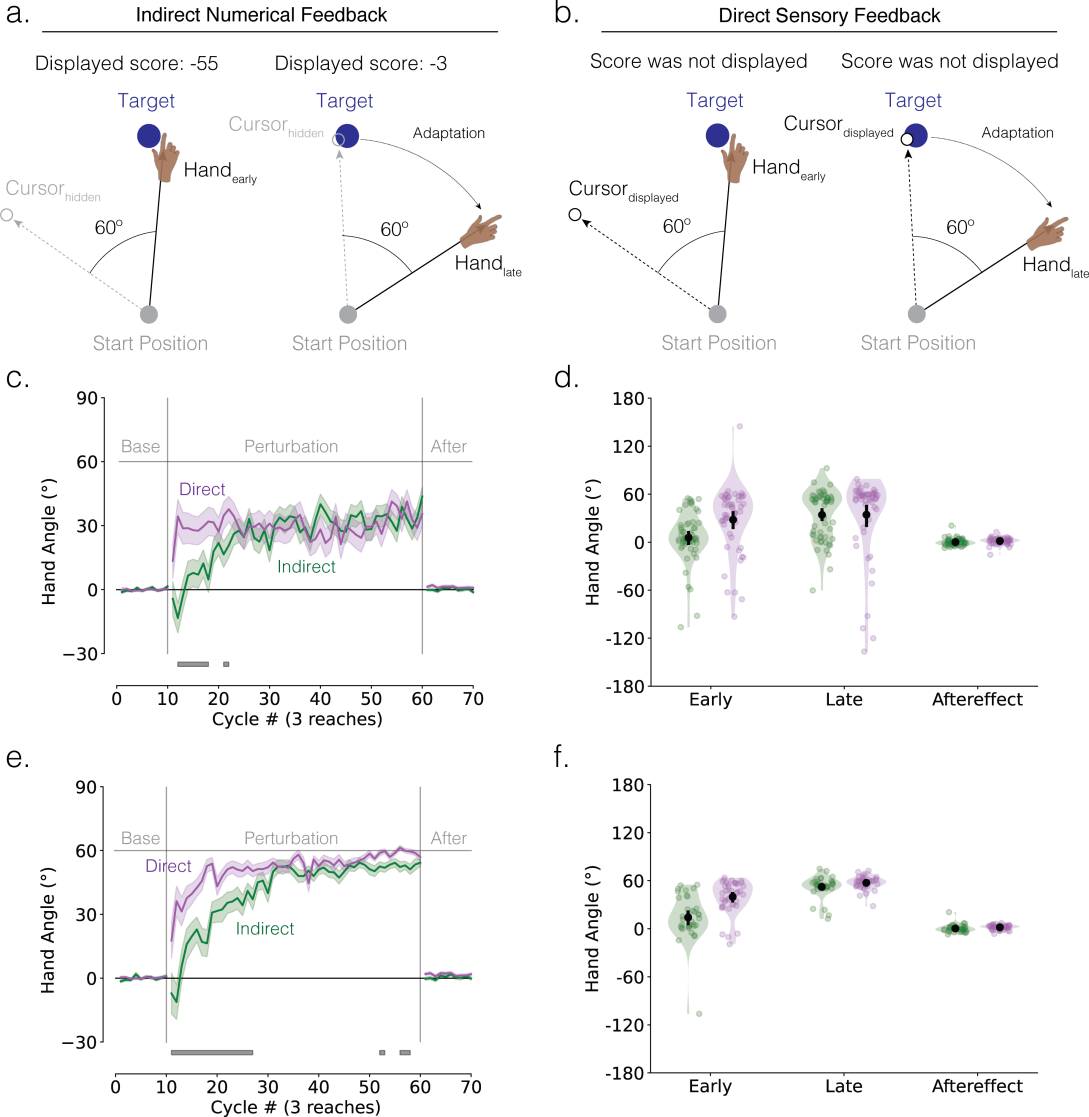
Indirect feedback decelerates explicit strategy use in response to a rotational perturbation. (a,b) Schematic of the 60° visuomotor rotation task (experiment 2). Feedback was delayed to minimize implicit recalibration. Participants were instructed to move in a direction that minimizes error. Error was conveyed through (a) indirect feedback (score magnitude conveys the size of the angular error rounded to the nearest integer; score sign conveys direction, with negative denoting a counterclockwise error and positive denoting a clockwise error) or (b) direct feedback (the magnitude and direction of the error are conveyed by a rotated cursor). Left and right panels denote early and late adaptation, respectively. (c) Mean time courses of hand angle (*n* = 110; direct: 53 participants; indirect: 57 participants). Colours denote different feedback groups (green = indirect feedback; purple = direct feedback). Shaded error denotes s.e.m. Grey horizontal line at the bottom indicates clusters showing significant group differences. (d) Mean hand angles during early adaptation, late adaptation and aftereffect phases. Black line denotes mean ± 95% confidence intervals. (e) Mean time courses and (f) mean hand angles of learners (*n* = 76; direct: 40 learners; indirect: 36 learners).

To provide a fair comparison between the two feedback types, we used perturbation conditions that should ensure learning is limited to explicit strategy use. To this end, we used a large perturbation (60° rotation) and presented the feedback 800 ms after the amplitude of the hand movement reached the target distance (figure 3*a–b*). The latter manipulation severely attenuates, or even eliminates any contribution of implicit recalibration [11,12]. Unlike experiment 1, the indirect feedback was modified to convey information about both error magnitude and error direction. In this way, we sought to create two conditions that only differed in whether the terminal position of the cursor was indicated by a direct cue at that position or indirect feedback indicating that position.

The learning functions for the indirect (green) and direct (purple) groups are shown in figure 3*c*. Both groups reached a similar level of asymptotic performance, one that fell short of counteracting the 60° perturbation. However, the direct group reached this asymptote within just a few movement cycles, a much faster rate than that exhibited by the indirect group. Both groups exhibited minimal aftereffects when the feedback was removed, confirming that the delayed feedback manipulation was successful in eliminating implicit recalibration.

These observations were verified statistically. First, the cluster-based permutation test demonstrated that the change in hand angle occurred more slowly in response to indirect feedback compared with direct feedback, with a significant difference between groups evident in many of the initial perturbation cycles (figure 3*c*; cluster-based *t*‐test: *t*_score_ > 4.11, *p*_perm_< 0.05, *d* > 0.22). This group difference was not significant after cycle 23. Second, both groups exhibited a large drop in hand angle after late adaptation (figure 3*d*; paired *t*-tests comparing aftereffect versus late adaptation: direct: −32.89 ± 6.84°, *t*(52) = −5.04, *p* < 0.001, *d* = −0.69; indirect: −34.06 ± 4.14°, *t*(56) = −6.81, *p* < 0.001, *d* = −0.90). Third, neither group exhibited a significant aftereffect (figure 3*d*; paired *t*-tests comparing aftereffect versus baseline: direct: 1.49 ± 0.56°, *t*(52) = −0.37, *p* = 0.716, *d* = −0.05; indirect: 0.11 ± 0.58°, *t*(56) = 0.18, *p* = 0.855, *d* = 0.02).

As in experiment 1, the low asymptotes for both groups were primarily due to fact that some participants in each group failed to come up with the correct strategy by the end of the experiment (direct: 25% non-learners; indirect: 37% non-learners). For the ‘learner’ subgroup, late adaptation in both feedback groups approximated the size of the perturbation (figure 3*f*; paired *t*-tests comparing late adaptation versus baseline: direct: 57.35 ± 1.26°, *t*(39) = 45.53, *p* < 0.001, *d* = 7.20; indirect: 52.30 ± 2.21°, *t*(35) = 23.62, *p* < 0.001, *d* = 3.94). Importantly, the cluster-based permutation test again demonstrated that learning occurred more slowly in response to indirect compared with direct feedback, with a significant hand angle difference between groups evident in both initial and late perturbation cycles (figure 3*e*; cluster-based *t*‐test: *t*_score_ > 4.41, *p*_perm_ < 0.05, *d* > 0.29).

There was a significant drop in mean hand angles in the aftereffect block after training (figure 3*f*: direct: −55.58 ± 1.29°, *t*(39) = −43.15, *p* < 0.001, *d* = −6.82; indirect: −51.93 ± 2.47°, *t*(35) = −20.99, *p* < 0.001, *d* = −3.50), with minimum aftereffect (figure 3*f*; paired *t*-tests comparing aftereffect versus baseline: direct: 1.78 ± 0.47°, *t*(39) = 3.82, *p* < 0.001, *d* = 0.60; indirect: 0.36 ± 0.81°, *t*(35) = 0.45, *p* = 0.659, *d* = 0.07).

Similar to experiment 1, we examined how feedback type influences exploration during early adaptation. The results closely mirrored our previous findings (figure 2*d–e*). First, learners within each group showed multimodal hand angle distributions (table 1). Second, both groups exhibited peaks around 0° (directly to the target), the correct solution at 60° and 180° (opposite to the target), along with a uniform component. This pattern reflects a combination of systematic and random exploration.

As reflected in the divergence measure, the prominence of different strategies varied by feedback type (JSD = 0.841, *p* = 0.002). The direct group showed a higher proportion of responses at the solution and fewer at the target, whereas the indirect group showed the opposite pattern. Moreover, the indirect group exhibited greater random exploration (direct: *w* = 0.00; indirect: *w* = 0.11) and more systematic exploration. The latter included a more prominent peak around −60°, a peak reflecting a sign-flip error—where participants reached in the wrong direction (electronic supplementary material, figure S1a).

The results of experiment 2 show that explicit motor adaptation is slower in response to indirect feedback compared to direct feedback even when implicit recalibration is minimized, and the error information is closely matched in terms of direction and magnitude. Indirect, abstract feedback hinders the discovery of a successful re-aiming strategy—as evidenced by increased systematic and random exploration.

### (c) Experiment 3: motor adaptation in response to a mirror-reversal perturbation is decelerated by indirect compared with direct feedback

**Figure 4.**
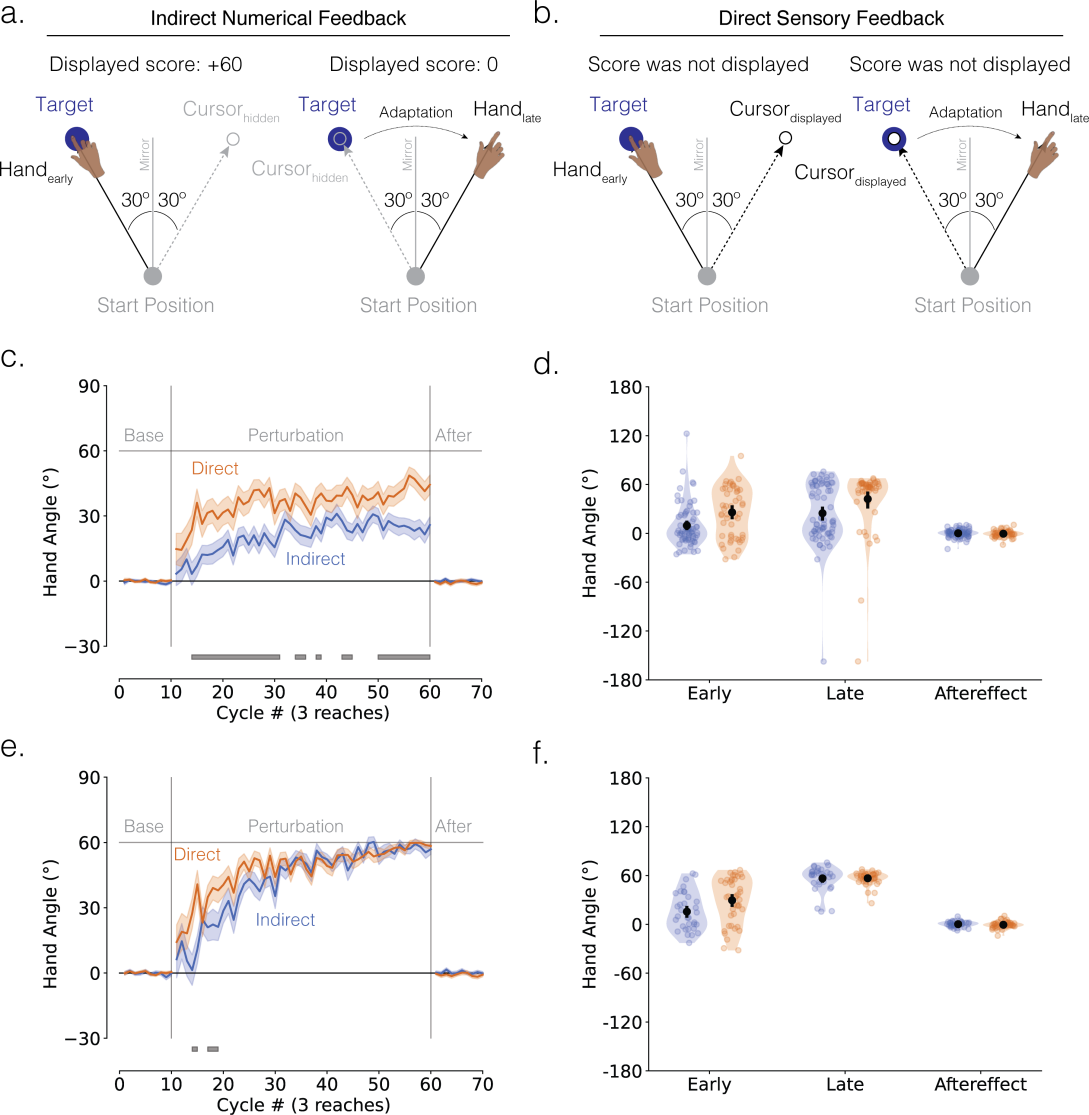
Indirect feedback decelerates explicit adaptation in response to a mirror perturbation. (a, b) Schematic of the mirror-reversal perturbation task in experiment 3. Participants were instructed to move in the direction that minimizes error. Error was conveyed through (a) indirect feedback or (b) direct feedback. Feedback is provided in the same manner as experiment 2. Left and right panels denote early and late adaptation, respectively. (c) Mean time courses of hand angle (*n* = 121; direct: 50 participants; indirect: 71 participants). Colours denote different feedback groups (royal blue = indirect feedback; orange = direct feedback). Shaded error denote s.e.m. Grey horizontal line at the bottom indicates clusters showing significant group differences. (d) Mean hand angles during early adaptation, late adaptation and aftereffect phases. Black line denotes mean ± 95% confidence interval. (e) Mean time courses and (f) mean hand angles of learners (*n* = 75; direct: 42 learners; indirect: 33 learners).

We designed experiment 3 to examine if the learning disadvantage observed with indirect numerical feedback would also be manifest with another type of perturbation. To test this, we used a mirror reversal of the visual feedback, a perturbation known to elicit adaptation mainly through explicit strategy use (figure 4*a–b*) [58–60].

When we compared the learning functions between the indirect feedback (royal blue) and direct feedback (orange) groups, our key results mirrored (pun intended) those of experiment 2 (figure 4*c*). The cluster-based permutation test revealed that the change in hand angle occurred slower in response to indirect feedback compared with direct feedback, with a significant difference between groups evident throughout the entire perturbation block (figure 4*c*; cluster-based *t*‐test, all participants: *t*_score_>4.94, *p*_perm_< 0.05, *d* > 0.25). Consistent with the assumption that performance on this task is strategy driven, both groups exhibited a significant drop in hand angle from the late adaptation to aftereffect phases (figure 4*d*; paired *t*-tests: direct: −42.71 ± 5.74°, *t*(49) = −6.30, *p* < 0.001, *d* = −0.89; indirect: −24.63 ± 4.47°, *t*(70) = −5.26, *p* < 0.001, *d* = −0.62). Neither group showed a significant aftereffect (figure 4*d*; paired *t*-tests against baseline: direct: −0.45 ± 0.58°, *t*(49) = −0.78, *p* = 0.441, *d* = −0.11; indirect*:* 0.12 ± 0.58°, *t*(70) = 0.20, *p* = 0.841, *d* = 0.02), suggesting that there was no implicit contribution to performance.

Unlike experiment 2, in the analysis, including all participants, the indirect group exhibited a markedly lower asymptote compared with the direct group. This is likely because there were significantly more non-learners in the indirect group compared with the direct group (*χ*^2^(1, *n* = 121) = 15.97, *p* < 0.001; direct: 16% non-learners; indirect: 54% non-learners). When we analysed only the data from learners, the difference between the two groups was much more subtle. Here, the indirect group showed a disadvantage only in the early phase of learning (figure 4*e*; learners only: *t*_score_>5.53, *p*_perm_<0.05, *d* > 0.24), with the two groups reaching a similar late level of late adaptation (figure 4*f*; paired *t*‐test comparing late adaptation versus baseline, direct: 56.62 ± 1.19°, *t*(41) = 47.68, *p* < 0.001, *d* = 7.36; indirect: 56.47 ± 2.60°, *t*(32) = 21.22, *p* < 0.001, *d* = 3.69). Both groups exhibited a large drop in hand angle from late to aftereffect phases (figure 4*f*; direct: −57.17 ± 1.17°, *t*(41) = −48.89, *p* < 0.001, *d* = −7.54; indirect: −56.25 ± 2.67°, *t*(32) = −20.77, *p* < 0.001, *d* = −3.61) and neither group exhibited an aftereffect (figure 4*f*; direct: −0.55 ± 0.65°, *t*(41) = −0.85, *p* = 0.402, *d* = −0.13; indirect: 0.21 ± 0.61°, *t*(32) = 0.35, *p* = 0.731, *d* = 0.06).

Our findings on strategy discovery across feedback types showed a similar pattern as observed in the previous experiments although the effects were less pronounced (JSD = 0.782, *p* = 0.19). Consistent with the slower learning observed in the hand angle data, the indirect group exhibited a higher proportion of responses at the target and fewer at the correct solution compared with the direct group (figure 2*f–g*; table 1). The indirect group also demonstrated more random exploration (direct: *w* = 0.06; indirect: *w* = 0.21) and greater and qualitatively different systematic exploration. In the direct group, alternative strategies were limited to subtle, short-lived peaks at 180° and 120°, the latter likely reflecting brief attempts to reach to the location of the other targets (figure 2*g*; electronic supplementary material, figure S1). In contrast, the indirect group showed sustained exploration of around the target location (figure 2*f*).

Additionally, we compared hand angle distributions among learners for rotation versus mirror reversal within experiments 2 and 3. Jensen–Shannon divergence analyses showed no significant differences within either the direct (JSD = 0.646, *p* = 0.144) or indirect feedback groups (JSD = 0.597, *p* = 0.905), indicating broadly similar exploration patterns regardless of perturbation types.

The results of experiment 3 reinforce the idea that indirect feedback impairs strategy discovery, as reflected in increased random and more divergent systematic exploration.

## 4. Discussion

In three well-powered studies, we examined how implicit and explicit learning processes are modulated by indirect feedback. Consistent with previous reports, we found that adaptation with indirect feedback was dominated by explicit strategy use, with minimal evidence of implicit recalibration. Moreover, even when the indirect feedback conveyed information content matched to that of direct feedback, adaptation was markedly slower due to increased random and systematic exploration. We postulate that the abstract and indirect nature of indirect feedback may impede learning by disrupting strategic reasoning and/or strategic refinement.

### (a) Motor adaptation in response to indirect feedback is dominated by an explicit re-aiming strategy

Building on measures that have been shown to be diagnostic of implicit and explicit processes in studies with direct feedback, the results of experiment 1 indicate that adaptation based on indirect feedback is dominated by explicit strategy use. Signatures of strategy use include the scaling of participants’ performance and reaction times with the size of the perturbations, along with negligible residual aftereffects once the perturbation was removed. These findings are consistent with previous research indicating that adaptation from indirect feedback arises from explicit strategy use [30].

The absence of aftereffects in our experiments is not a limitation of the online, web-based method. Prior studies using our platform have reliably replicated classic learning patterns and robust aftereffects in standard visuomotor rotation tasks [10,14]. In experiment 1, the lack of aftereffects seems to reflect a genuine absence of implicit recalibration in response to indirect feedback. In the direct feedback group, aftereffects were intentionally minimized by imposing a delay—allowing us to isolate explicit strategy use and equate the learning process across feedback types in experiments 2 and 3 [11–13,15,16] (see electronic supplementary material, Supplemental Discussions I and II for further elaboration).

### (b) Explicit adaptation is decelerated by indirect feedback, driven by increased exploratory behaviour

The results of experiments 2 and 3 showed that learning was impeded by indirect compared with direct feedback. Below, we consider three potential reasons. First, we hypothesize that the abstract nature of indirect feedback impairs learning by disrupting reasoning, the process of understanding action–outcome relationships. That is, if indirect feedback impairs reasoning about the nature of the perturbation, it should lead to broader, slower hypothesis testing during the early strategy discovery phase. Consistent with this prediction—and particularly evident in experiment 2—indirect feedback elicited greater systematic exploration, as reflected in more distinct peaks in hand angle distributions.

Second, feedback type may engage different forms of reasoning. Specifically, we observed greater random exploration under indirect feedback, indicated by increased weight on the uniform component of our mixture model. This may reflect a difference in hypothesis-driven reasoning and a more heuristic, trial-and-error process (e.g. win–stay, lose–shift). With direct feedback, participants’ hand angle distributions were narrowly centred around discrete action–outcome hypotheses. This could be due to the immediate spatial information about movement errors afforded by direct sensory feedback, enabling participants to test candidate perturbation types (e.g. rotation versus reversal) and rapidly update their beliefs.

In contrast, indirect feedback provides abstract, numerical information divorced from spatial context, increasing uncertainty about the size and direction of the perturbation. As a result, participants may struggle to form accurate hypotheses and instead rely on slower, less structured forms of reasoning—repeating successful actions and avoiding unsuccessful ones [24,61]. Future work could distinguish between these mechanisms by probing generalization: hypothesis-based learning should generalize to novel target locations, whereas trial-and-error learning would yield more limited, target-specific generalization locations [42].

Third, indirect and direct feedback may differ in how they support refinement—the process of optimizing sensorimotor or cognitive parameters to achieve a motor goal. By this view, learners in both conditions may discover the nature of the perturbation at a similar rate, but those receiving direct feedback—who had immediate visual information about the size and direction of the error—may more easily convert that understanding into an accurate motor plan. In contrast, the abstract nature of indirect feedback may make it harder to translate this knowledge into an effective control policy, thereby slowing strategic refinement. Together, our findings suggest that feedback influences how strategies are *discovered* and/or *refined*.

## Data Availability

Raw data and analysis code in Python can be openly accessed at OSF [62]. Supplementary material is available online [63].
